# p53 protects against formation of extrahepatic biliary precancerous lesions in the context of oncogenic Kras

**DOI:** 10.18632/oncotarget.28380

**Published:** 2023-03-31

**Authors:** Munemasa Nagao, Kenta Mizukoshi, Shinnosuke Nakayama, Mio Namikawa, Yukiko Hiramatsu, Takahisa Maruno, Yuki Nakanishi, Tatsuaki Tsuruyama, Akihisa Fukuda, Hiroshi Seno

**Affiliations:** ^*^These authors contributed equally to this work

**Keywords:** BilIN, ICPN, biliary cancer, Kras, p53

## Abstract

*KRAS* and *TP53* mutations are frequently observed in extrahepatic biliary cancer. Mutations of *KRAS* and *TP53* are independent risk factors for poor prognosis in biliary cancer. However, the exact role of p53 in the development of extrahepatic biliary cancer remains elusive. In this study, we found that simultaneous activation of Kras and inactivation of p53 induces biliary neoplasms that resemble human biliary intraepithelial neoplasia in the extrahepatic bile duct and intracholecystic papillary-tubular neoplasm in the gall bladder in mice. However, inactivation of p53 was not sufficient for the progression of biliary precancerous lesions into invasive cancer in the context of oncogenic Kras within the observation period. This was also the case in the context of additional activation of the Wnt signaling pathway. Thus, p53 protects against formation of extrahepatic biliary precancerous lesions in the context of oncogenic Kras.

Biliary tract cancer (BTC) includes cholangiocarcinoma (CCA) and gall bladder carcinoma (GBC). According to the World Health Organization (WHO) databases, the global mortality for CCA increased worldwide [1]. The 5-year survival rate of biliary cancer remains only 5% to 15% [2, 3]. Recently, biliary intraepithelial neoplasm (BilIN) and intraductal papillary neoplasm of the bile duct (IPNB) were defined as precursor lesions of invasive adenocarcinoma by the WHO classification [4–6]. BilIN is defined as a microscopically identifiable, pre-invasive neoplastic lesion of the biliary lining epithelia of the bile duct. IPNB is defined as a grossly visible, intraductal, preinvasive papillary or villous epithelial neoplasm covering fine fibrovascular stalks [7]. Papillary lesion like IPNB in the gall bladder (GB) is intracholecystic papillary-tubular neoplasm (ICPN) [8]. However, the molecular mechanism of formation of these precursor lesions is not fully understood.

Whole-genome sequencing (WGS) studies have revealed high incidence of mutations in *KRAS* (17–18%) and *TP53* (26–32%) in biliary cancer [9–11]. The incidence of *KRAS* mutation and *TP53* mutation is 31.5% and 36.9%, respectively, in extrahepatic cholangiocarcinoma [12]. Mutations of *KRAS codon12* occurred in about 50% of early BilIN [13]. These findings suggest the possible roles of mutations in Kras and p53 in biliary tumorigenesis.

To clarify the exact role of each molecule in which mutations are observed in biliary cancer, genetically engineered mouse model (GEM) is a powerful tool. Recently, we have shown that *Hnf1b^CreER^* mouse is one of the useful biliary-specific driver CreER mouse lines for gene manipulation in the extrahepatic biliary duct (EHBD) and GB, and that concurrent activation of the Kras and canonical Wnt pathways in the adult biliary epithelial cells induces BilIN and ICPN in the EHBD and GB, respectively [14].

It is well-known that p53 deficiency promotes the progression of precancerous lesions into invasive adenocarcinoma in the context of oncogenic Kras in the pancreas [15]. Therefore, we hypothesized that inactivation of p53 also promotes tumorigenesis in the extrahepatic biliary system. To determine whether mutation of *KRAS* or *TP53* affects the progression of human cholangiocarcinoma, we first performed prognosis analyses of cholangiocarcinoma using the The Cancer Genome Atlas (TCGA) database. Overall survival was compared among the group with mutation of *KRAS*, group with mutation of *TP53*, and group without *KRAS* or *TP53* mutation. Mutations of *KRAS* and *TP53* were each negatively correlated with overall survival in human biliary cancer patients (Figure 1A). Additionally, overall survival was comparable between the group with mutation of *KRAS* and *TP53* and the group without mutation of *KRAS* and *TP53*. These data indicated that mutations of *KRAS* and *TP53* were independent risk factors for poor prognosis in human biliary cancer.

**Figure 1:**
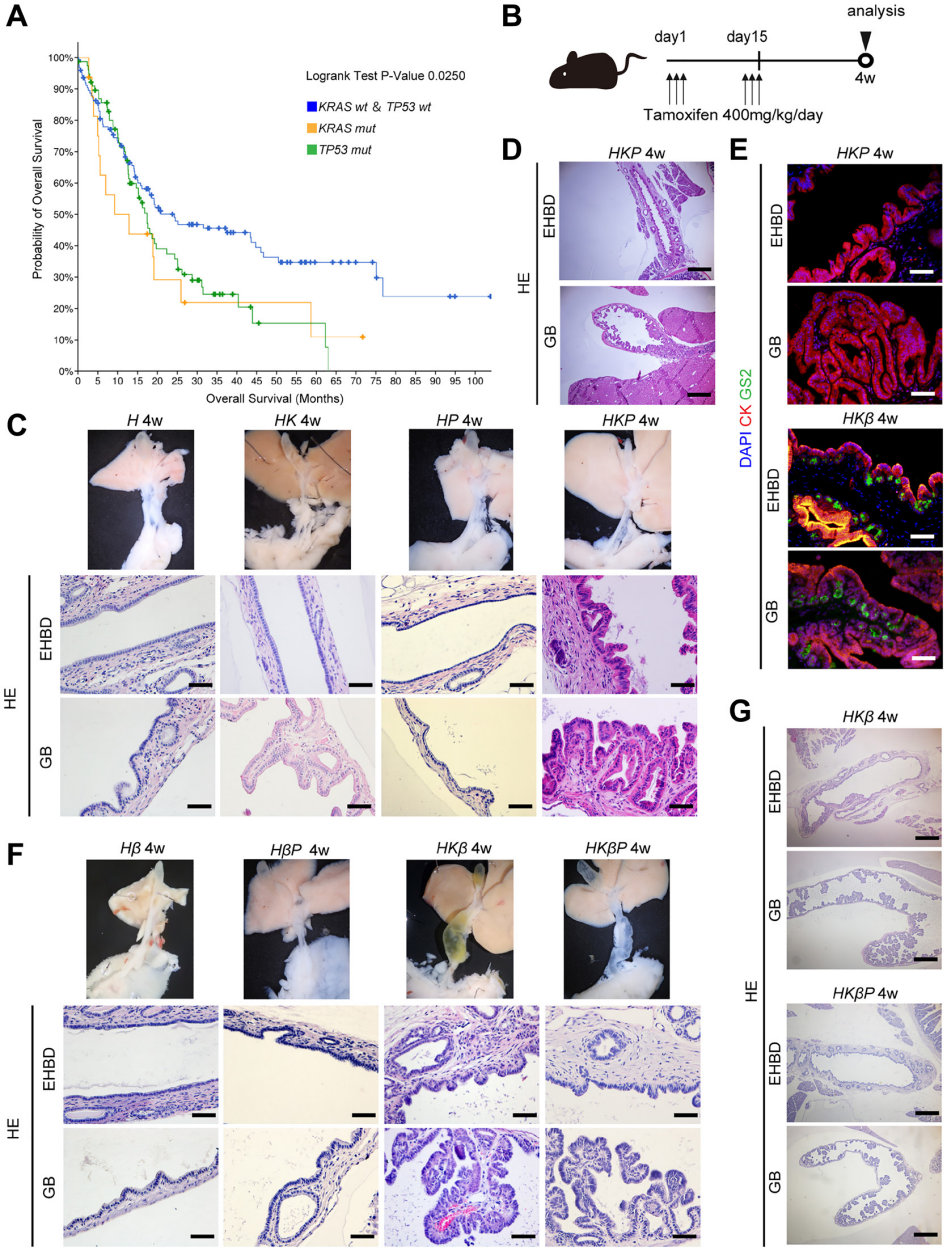
Simultaneous activation of Kras and inactivation of p53 induced ICPN and BilIN that resemble human ICPN and BilIN. (**A**) Kaplan-meier curve of overall survival for group with mutation of *KRAS* and *TP53* and group without mutation of *KRAS* or *TP53*. (**B**) Tamoxifen administration schema for the experiments using *Hnf1b^CreER^*-line mice. All mice were sacrificed 4 weeks after the last tamoxifen administration. (**C**) Macroscopic (upper) and microscopic (lower) images of the EHBD and GB in *H, HK, HP*, and *HKP* mice 4 weeks after the last tamoxifen administration. (black scale bars = 50 μm). (**D**) Low-power field of microscopic images of the EHBD and GB in *HKP* mice 4 weeks after the last tamoxifen administration (black scale bars = 500 μm). (**E**) Coimmunostaining for DAPI (blue), GS-II (green), and CK19 (red) in the EHBD and GB in *HKP* and *HKβP* mice 4 weeks after the last tamoxifen administration. (**F**) Macroscopic (upper) and microscopic (lower) images of the EHBD and GB in *Hβ, HKβ, HβP,* and *HKβP* mice 4 weeks after the last tamoxifen administration. (All black or white scale bars = 50 μm). (**G**) Low-power field of microscopic images of the EHBD and GB in *HβP* and *HKβP* mice 4 weeks after the last tamoxifen administration. (black scale bars = 500 μm).

We next investigated the functional role of Kras and p53 in tumorigenesis of the biliary system using the *Hnf1b^CreER^* mouse line [14]. We crossed *Kras^G12D^* mice and/or *Tp53^flox/flox^* mice with *Hnf1b^CreER^* mice to generate *Hnf1b^CreER^(H), Hnf1b^CreER^*; *Kras^G12D^ (HK), Hnf1b^CreER^*; *Tp53^flox/flox^ (HP)*, and *Hnf1b^CreER^*; *Kras^G12D^*; *Tp53^flox/flox^ (HKP)* mice. Four weeks after the last tamoxifen administration, the biliary tract was analyzed (Figure 1B). Macroscopically, the EHBD in *H*, *HK, HP*, and *HKP* mice looked normal (Figure 1C). H&E staining revealed an almost normal appearance of the GB and EHBD epithelial cells in *H*, *HK* and *HP* mice. In contrast, *HKP* mice displayed microscopic papillary neoplasms which resembled human BilIN in the EHBD and papillary neoplasm which resembled human ICPN in the GB (Figure 1C, 1D). Neoplastic changes were also observed in the peribiliary glands of the EHBD in *HKP* mice. Hyperchromasia, nuclear stratification, and partial loss of nuclear polarity were observed in the epithelial cells of the BilIN and ICPN lesions in *HKP* mice. Immunohistochemistry (IHC) for mucin was next performed to assess the subtypes of BilIN and ICPN in *HKP* mice. Muc1 was positive in biliary epithelial cells in *HKP* mice, whereas muc2 and muc5AC were negative. In our previous report, Griffonia simplicifolia lectin II (GSII lectin) was useful as an alternate marker for muc6 in mice [14]. Staining of GSII revealed that GSII was not expressed in BilIN and ICPN in *HKP* mice (Figure 1E). In our recent report, concurrent activation of the Kras and canonical Wnt pathways induces GSII-positive or gastric BilIN and ICPN (Figure 1E). In contrast, BilIN and ICPN in *HKP* mice did not represent the gastric type. These data suggested that activation of the Wnt pathway induces biliary precancerous lesions into the gastric type, whereas p53 inactivation does not. These data indicated that concurrent activation of Kras and inactivation of p53 induces BiliN in the EHBD and ICPN in the GB in *HKP* mice. However, inactivation of p53 was not sufficient for the progression of precancerous lesions into adenocarcinoma in the extrahepatic biliary system within the observation period. Longer-term analysis was not possible, because *HKβ* and *HKβP* mice died due to lung cancers at 6 to 8 weeks of age after tamoxifen treatment.

We next investigated whether inactivation of p53 promotes biliary precancerous lesions into adenocarcinoma in the context of activated Kras and Wnt signaling in mice. To this end, we crossed *Kras^G12D^* mice and/or *Tp53^flox/flox^* mice and *Ctnnb1^lox(ex3)/+^* mice with *Hnf1b^CreER^* mice to generate *Hnf1b^CreER^*; *Ctnnb1^lox(ex3)/+^ (Hβ)*, *Hnf1b^CreER^*; *Ctnnb1^lox(ex3)/+^ Tp53^flox/flox^ (HβP)*, *Hnf1b^CreER^*; *Kras^G12D^ Ctnnb1^lox(ex3)/+^ (HKβ)*, and *Hnf1b^CreER^*; *Kras^G12D^*; *Ctnnb1^lox(ex3)/+^*; *Tp53^flox/flox^ (HKβP)* mice. Four weeks after the last tamoxifen administration, the biliary tract was analyzed (Figure 1B). Macroscopically, the EHBD in *Hβ* and *HβP* mice appeared normal, whereas the EHBD was dilated in *HKβ* and *HKβP* mice. H&E staining revealed an almost normal appearance of the GB and EHBD epithelial cells in *Hβ* and *HβP* mice. In contrast, *HKβ* and *HKβP* mice displayed microscopic papillary neoplasms, which resembled human BilIN in the EHBD and ICPN in the GB (Figure 1F, 1G). However, neoplastic grade of BilIN and ICPN was not different between *HKβ* and *HKβP* mice. These data indicated that inactivation of p53 did not accelerate the progression of biliary precancerous lesions into adenocarcinoma even in the context of activated Kras and Wnt signaling. Longer-term analysis was not possible, because *HKβ* and *HKβP* mice died due to massive intestinal adenomas at 6 to 8 weeks of age after tamoxifen treatment.

In conclusion, p53 protects against formation of extrahepatic biliary precancerous lesions in the context of oncogenic Kras in mice, however, inactivation of p53 is not sufficient for the progression into invasive cancer in the extrahepatic biliary system.

## Abbreviations

BilIN: biliary intraepithelial neoplasia
BTC: biliary tract cancer
CCA: cholangiocarcinoma
EHBD: extrahepatic bile duct
GB: gall bladder
GBC: gall bladder carcinoma
GEMs: genetically engineered mouse models
ICPN: intracholecystic papillary-tubular neoplasm
IHC: immunohistochemistry
TCGA: The Cancer Genome Atlas
WGS: whole-genome sequencing
WHO: World Health Organization
